# Effect of cellulose nanofibers on the fracture toughness mode II of glass fiber/epoxy composite laminates

**DOI:** 10.1016/j.heliyon.2023.e13203

**Published:** 2023-01-22

**Authors:** Mouhamadou Moustapha Sarr, Tatsuro Kosaka

**Affiliations:** a Department of Science and Industrial Techniques, Ecole Normale Supérieure d’Enseignement Technique et Professionnel, Cheikh Anta Diop University, BP5004, Dakar, Senegal; b Department of Engineering, Kochi University of Technology, 782-8502, Kami-shi, Japan

**Keywords:** Glass fiber reinforced polymer, Cellulose nanofibers, Interlaminar fracture toughness, End notch flexural test

## Abstract

Cellulose nanofibers (CNFs) were used to improve the fracture toughness of glass fiber reinforced epoxy composites (GFRPs). Different CNF suspensions were prepared and sprayed onto the surface of woven glass fiber laminates. The vacuum resin transfer molding (VaRTM) process was used to manufacture the GFRP composites. End notch flexure tests were conducted to evaluate the effect of CNFs on the critical energy release rate in mode II fracture toughness G_IIC_. The results revealed that 0.05 wt% was the optimum concentration. The interlaminar fracture toughness G_IIC_ was improved by 28% with the addition of 0.05 wt% of CNFs to GF/epoxy composites. Whereas 0.1 wt% of CNFs resulted in decreasing G_IIC_ due to the uncomplete impregnation of GF with epoxy resin caused by the thicker CNF layer at the interfacial laminates. The toughening mechanisms were investigated using a field-emission electron microscope. Large epoxy deformations and shear hackles were predominant for improving the interlaminar fracture toughness of GFRP composites.

## 1 Introduction

Glass fiber reinforced polymer (GFRP) composites are widely used as engineering materials in various industries (e.g., aerospace, automotive, marine) due to their combination of excellent mechanical, chemical, and physical properties. The need for efficient fuel consumption and lightweight structures in industrial applications has grown the use of fiber-reinforced polymers (FRP) composites. Thermoset polymers such as epoxy resins are commonly used in GFRP composites owing to their relatively high strength, good chemical resistance, low cost, excellent thermal stability, and good flexibility. However, they become brittle after being cured with crosslinked agents, making composites sensitive to damage, including crack initiation and propagation.

Cracks propagate under a repetitive or constant load leading to delamination, thus, bringing about fracture of composite materials. Delamination or interlaminar failure occurs at the resin-rich interlayer between two reinforcing plies mainly undergoing tensile mode (mode I), shear mode (mode II), or tearing mode (mode III) loading conditions [1]. The resistance of composite materials to these failures is interlaminar fracture toughness (IFT) or critical energy release rate (G_C_). The IFT mode II (G_IIC_) is one of the most prevalent loading modes in composite laminates, especially for aerospace applications. This mode is also known as rotation mode, occurring under flexural loads. Hence, interlaminar fracture toughness is a critical parameter when designing fiber-reinforced polymer (FRP) composites because a low IFT leads to failure in the entire composite. Due to high shear and peeling stress concentrations, delamination of composite laminates might occur, and fracture toughness under shear loading is a significant interface parameter to evaluate possible crack propagation [2]. Thus, it is necessary to improve the G_IIC_ of FRP composites through resin toughness at the resin-rich regions where delamination takes place. Various methods such as incorporating nanofillers into the matrix [3–5], modifying the structure of the fiber preforms by Z-pinning [6] or stitching [7], and interlaying composite laminates with interleaved nanofibers [8,9] or thermoplastics [10] have been used to improve the interlaminar fracture toughness of composite laminates.

In the last two decades, many research studies have been conducted using nanomaterials to enhance the IFT of FRP composites. Carbon nanotubes (CNT) [4,11], silica-nanoparticles [5], nanoclay [12] were incorporated into different matrices to improve the fracture toughness of FRP composites. Quan et al. [11] demonstrated that incorporating 1 wt% of multiwall carbon nanotubes (MWCNT) into the matrix of carbon fiber/epoxy composites improved G_IIC_ significantly by 170% resulting from MWCNT breakage and crack deflection under shear load; while the IFT mode I (G_IC_) exhibited moderate enhancement. Similarly, an increase of 27% in G_IC_ with 0.5 wt% of MWCNT modified epoxy nanocomposites was reported by Saboori et al. [13], while it decreased slightly with 1 wt% of MWCNT content. Seyhan et al. [4] prepared a matrix resin containing 0.1 wt% of functionalized CNTs using the three-roll milling method. After manufacturing the composite laminates by vacuum-assisted resin transfer molding (VaRTM), the GF/MWCNT modified resin exhibited an 11% improvement in fracture toughness mode II, while mode I showed a decrease. However, this toughening method may be limited due to the increase in epoxy viscosity which should not exceed the critical viscosity for epoxy infusion processes. Moreover, it is reported that during matrix infusion by VaRTM and curing process, CNTs are filtrated, indicating their inhomogeneous dispersion in the composite [14].

Many studies have focused on the interlaying method to avoid the drawbacks mentioned previously. Electrospun thermoplastic nanofibers used as an interlayer at the midplane of FRP composite laminates have significantly improved interlaminar fracture toughness [15]. Daelemans et al. [1] studied the toughening mechanism in carbon fiber/epoxy composite by interleaving polyamide (PA) nanofiber veils. They reported a maximum of 42% and 190% improvements in both G_IC_ and G_IIC_, respectively. The noteworthy increase in mode II might be due to a good load transfer to nanofibers along fiber direction arising from crack propagation and shear stresses. Shin et al. [16] incorporated high concentrations of CNTs into epoxy using ultrasonication and three-roll milling to make CNTs/epoxy film-interleave, which were used to improve the fracture toughness of CFRP composites. This method increased the G_IIC_ of the composites up to ∼ 127% with 3 wt% CNTs/epoxy film-interleaved. Moreover, Mirjalili et al. [14] investigated the effect of MWCNT as a toughening agent of CFRP composites by resin film infusion (RFI). They prepared two modified resin film systems: (1) with 0.3 wt% MWCNTs + resin/hardener, and (2) with 0.3 wt% MWCNTs + thermoplastic + resin/hardener. Significant improvements of 106% and 108% in G_IC_ and G_IIC_, respectively, were observed with the composite manufactured with the (2) resin film system. Based on this literature review, the effect of nanomaterials on the interlaminar fracture toughness of FRP laminates is much more effective in G_IIC_ than in G_IC_.

Recently, cellulose nanofibers (CNFs) have become a great interest for many researchers to improve the mechanical performance of FRP composites due to their high specific strength and stiffness, low density, and being environmentally friendly [17]. CNFs are prepared from different biological sources such as wood, bacteria, tunicate, cotton, manila. In addition, CNF is an attractive material for engineering and composites owing to its abundance of sources, thermal stability, and renewability [18]. However, the hydrophilicity of CNFs makes them difficult to disperse uniformly in most polymeric matrices. To increase the hydrophobicity and compatibility of CNFs with polymers, different chemical modifications on the surface of CNFs, including acetylation [19,20] and silane coupling [21] have been used.

In recent years, other techniques, including coating CNFs on reinforcing fibers or applying CNF sheets using electro-activated deposition resin molding (ERM), have been developed [22]. Katagiri et al. [22] improved significantly mechanical properties of CFRP composites by applying CNF sheets on the surface of the composites. Uribe et al. [23] coated CNFs on the surface of CF by immersion and spraying methods. The results showed that spraying CNFs was the most effective method to improve the mechanical performance of CF/Epoxy composites. This method increased tensile strength and tensile toughness up to 28% and 52%, respectively, higher than those from the immersion method. In our previous study [24], we developed a new approach of grafting CNFs on the surface of the reinforcing fibers by vacuum impregnation using the VaRTM technique. Subsequently, improvements of 78% and 20% were observed in the interfacial shear strength (IFSS) and flexural strength of GFRP composites, respectively. Nevertheless, the downside of this approach is the non-uniform dispersion of CNFs throughout the woven GF laminates due to the filtering effect of the reinforcing fibers. Zhu et al. [8] used waterborne epoxy with CNFs to manufacture CNF interleaves by freeze-drying. After that, CNFs interleaves were inserted between layers of CFRP laminates. The results revealed 22% and 25% improvements in G_IC_ and G_IIC,_ respectively, by adding a small quantity of CNFs. However, there are very limited studies on the effect of CNFs on interlaminar fracture toughness.

Based on the literature review, no studies have reported the effect of CNF grafting on reinforcing fibers on the interlaminar fracture toughness of FRP composite laminates. Therefore, in this study, a new simple method of modifying the structure of the interlaminar interface of GF laminates was proposed to improve the interlaminar fracture toughness. CNF suspensions with different concentrations (0.05, 0.075, and 0.1 wt%) were applied to the interlaminar interfaces of woven GF laminates. The ability of CNFs to withstand crack propagation and delamination of GFRP composites was investigated. The critical energy release rate for IFT mode II (G_IIC_) was evaluated by conducting three-point end notch flexure (ENF) tests. To clarify the toughening mechanisms, the morphology of fracture surfaces of GFRP composites after ENF tests was examined using a field-emission electron microscope (FE-SEM).

## 2 Experimental methods

### 2.1 Materials

In this study, woven glass fiber was supplied by Nittobo Techno Co. (Japan) with an areal density of 104 g/m^2^. A low viscosity epoxy-phenol novolac resin (Araldite LY5052) and cycloaliphatic polyamine (Aradur 5052) used as curing agents, were purchased from Huntsman Advanced Materials (Switzerland). The mixing ratio recommended by the supplier was 100:38 by weight. CNF was received from Kochi Prefectural Paper Industry Technology Center (Japan) in the slurry form with 2 wt% of CNF content. CNF is produced from wood with a width estimated between 10 and 50 nm. The length of CNFs is not revealed by the manufacturer [25].

### 2.2 Preparation of woven GFs with CNFs

CNFs were mixed with purified water and ultrasonicated using an MCS-10 As-One to prepare three CNF suspensions with different weight fractions (0.05, 0.075, and 0.1 wt%). Ultrasonication was used to disaggregate and disperse CNFs by hydrodynamic shear force. On the other hand, woven glass fibers were burnt out in a furnace at 350 °C for 1 h to remove the organic sizing agent. After cooling them down in the furnace, woven GFs were successively washed with acetone, isopropanol, and purified water. The washed woven GFs were then dried in an air oven at 60 °C for 3 h. The details of CNFs and woven GFs preparations are clearly described in our previous study [24].

### 2.3 Manufacturing of GFRP composites

**Fig. 1:**
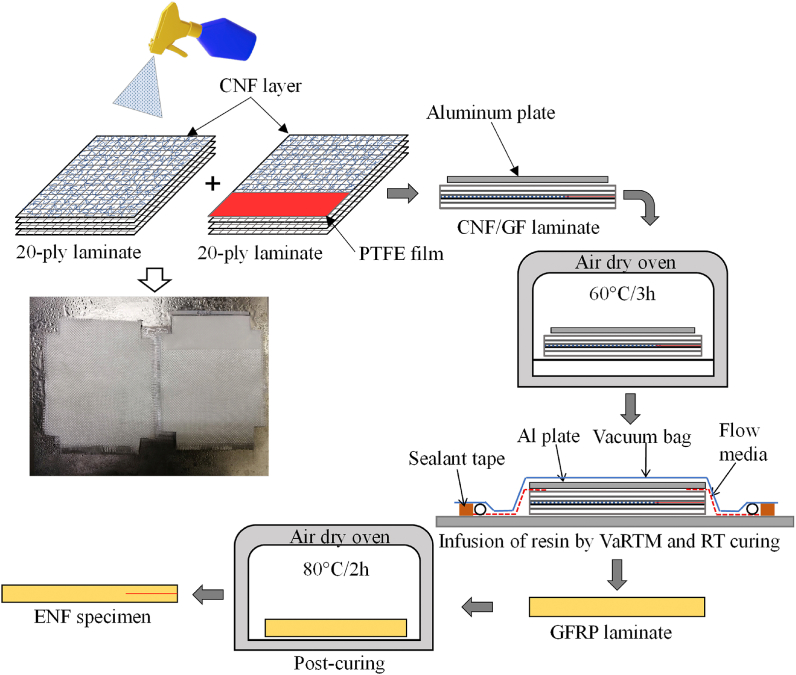
Illustration of CNF incorporating GF and manufacturing GFRP composite laminates.

Forty GF plies (135 × 105 mm^2^) were cut from unsized woven GFs, and then laid up in two laminates of twenty plies each. CNF suspensions were then sprayed three times onto the top surface of each 20-ply laminate using a manual spray-gun (Trusco, TSG-500G). After spraying CNFs, a 50 μm PTFE film with 40 mm width was placed at one end of the 20-ply laminate to form an initial crack. The laminates were stacked on each other and then dried in the air oven at 60 °C for 5 h. Before the drying process, an aluminum plate (125 × 100 × 5 mm³) was placed on the 40-ply laminate and moderately pressed to ensure enough contact between the CNFs treated surface of each laminate. Hence, the VaRTM process was used to infuse epoxy resin (Epoxy 108.7 g + curing agent 41.03 g) into the laminate. Before its use, epoxy resin (EP) was mixed and then degassed to remove air bubbles using a vacuum desiccator. The laminated composite was cured at room temperature (RT) for 20 h, followed by post-curing at 80 °C for 2 h in the oven. The volume fraction of the GFRP composite laminates was approximately ∼ 40%. Three specimens were cut from each composite laminate for ENF testing. Fig. 1 illustrates the different stages of the manufacturing process for GFRP composite laminates.

### 2.4 End notched flexure tests

**Fig. 2:**
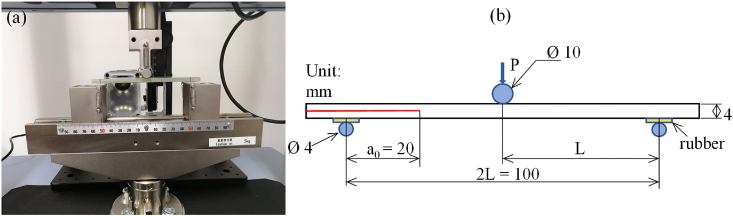
Three-point ENF test specimen set-up.

**Fig. 3:**
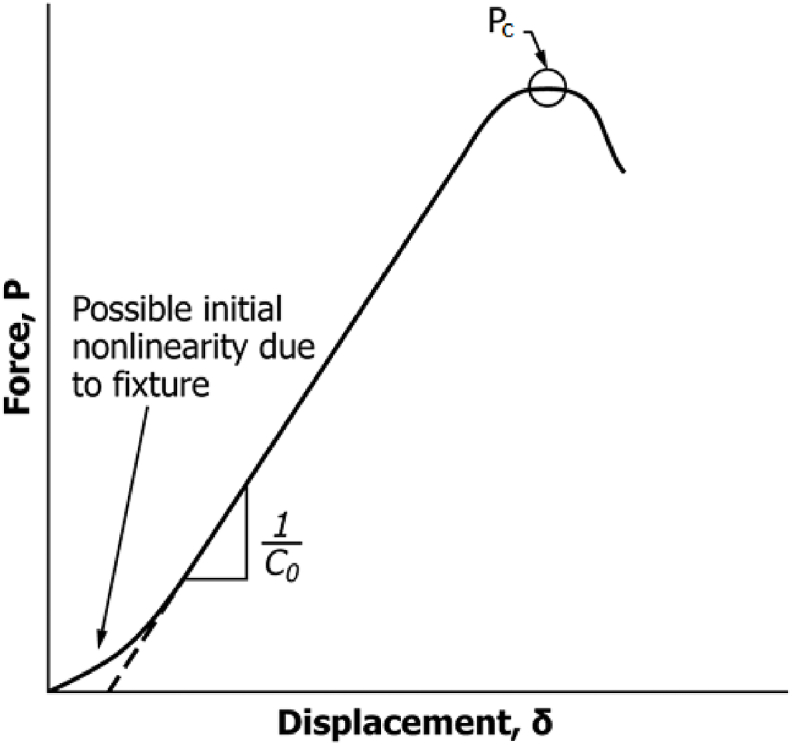
Illustration of compliance and critical load determination.

ENF tests were performed using a 10 kN Shimadzu Autograph AGS-X (Japan) testing machine to evaluate the resistance of interlaminar fracture toughness G_IIC_ of the GFRP composites according to the JIS K7086 [26]. It is not necessary to track crack propagation during the test when using this standard procedure, which could demand the use of additional equipment. Fig. 2a shows the setup of ENF test with the specimen. As illustrated in Fig. 2b, the specimens were cut from the composite laminates (120 × 25 × 4 mm³) and positioned at a constant distance of 20 mm between the PTFE film tip and the center of the supporting roller. The load (P) and displacement (δ) were controlled at a crosshead speed of 2 mm/min and recorded with a non-contact video type extensometer (TRAPEZIUM Lite X, Shimadzu, Japan). The interlaminar fracture toughness of GFRP composites was determined by calculating the critical energy release rate G_IIC_ using the following equations (1) and (2) [26]:

(1) $$G_{IIC}=\frac{9{.P}_{1}^{2}.a_{1}^{2}.C_{1}}{2B\left(3a^{3}+2L^{3}\right)}$$

(2) $$a_{1}={\left[\frac{C_{1}}{C_{0}}\;a_{0}^{3}+\frac{2}{3}\left(\frac{C_{1}}{C_{0}}-1\right)L^{3}\right]}^{\frac{1}{3}}$$

where $P_{1}$ indicates the critical load, which is determined from the load-displacement data. $a_{1}$ and $a_{0}$ are the crack length at the critical load $P_{1}$ and the initial crack length, respectively. $C_{1}$ ($\delta_{1}/P_{1}$) indicates the compliance at the critical load, and $C_{0}$ indicates the compliance within the linear elastic deformation before crack propagation (see Fig. 3). The crack length at the critical load $a_{1}$ was determined using the measured compliance $C_{1}$. In this study, $C_{0}$ was determined using the data ranging from 300 to 500 N L and B represent half-length of the span and the width of the specimen, respectively. At least three specimens were tested for each condition of composite laminates. The first test failed because the specimen slipped under a relatively high bending load. Therefore, silicon rubber with 2 mm in thickness was placed on the underside of the specimen above the supporting rollers. The initial nonlinear part of P vs. δ curves due to the high deformability of silicon was not considered for the calculation.

The morphology of CNFs-treated woven GF (GF-CNFs) laminates and the fracture surfaces of the GFRP composites after ENF test was examined to investigate the toughening mechanism using an FE-SEM (Hitachi SU8020, Japan). A low accelerating voltage of 1.5 kV was selected during the observation to avoid the metal coating sputter, which may change the specimen's morphology. Low accelerating voltages are recommended for non-conductive or beam-sensitive specimens [27].

## 3 Results and discussion

### 3.1 Morphology of the CNFs-treated woven GF laminates

**Fig. 4:**
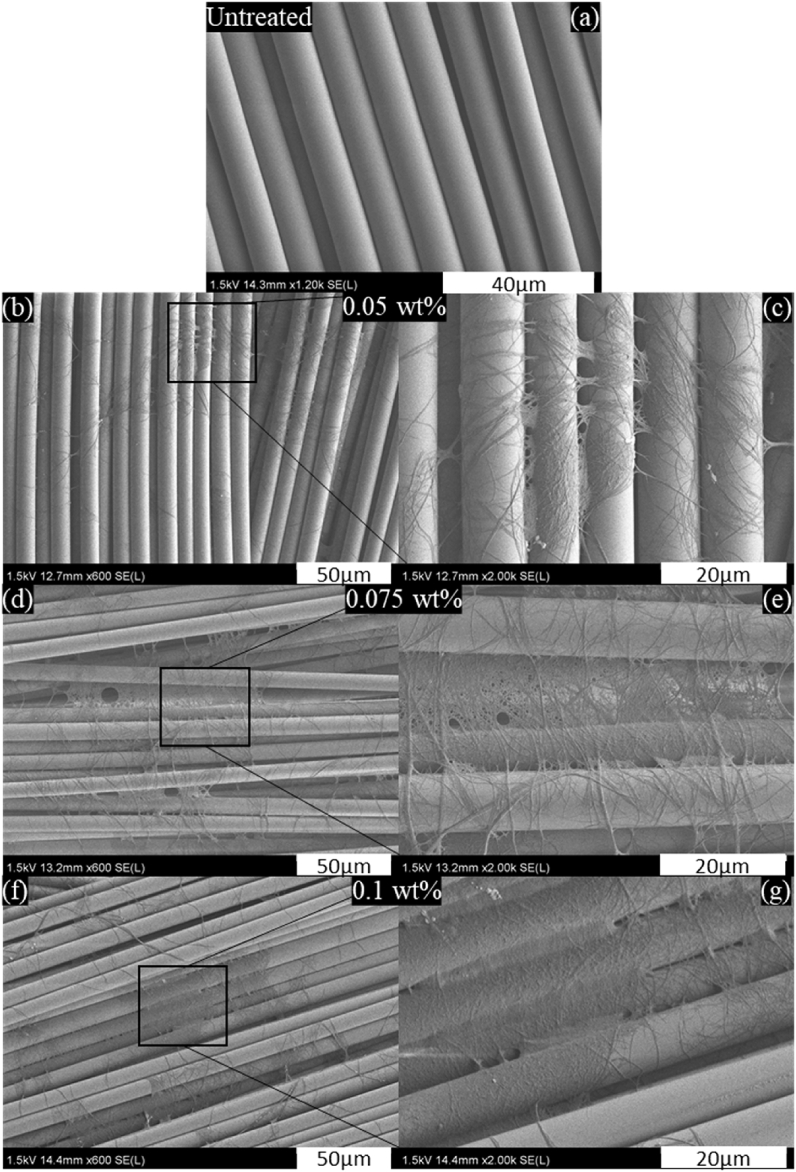
Morphology of untreated woven GFs laminate (a), and CNFs treated woven GFs laminates: 0.05 wt% (b–c), 0.075 wt% (d–e), 0.1 wt% (f–g).

Fig. 4 characterizes the morphology of woven GFs after spraying CNFs and drying the reinforced laminates. It can be observed that CNFs were randomly coated on the GF surface (Fig. 4b, d, f). Regardless of the CNF content, a web-like nanostructure is formed on the surface of GF laminates and bridged between fibers (Fig. 4c, e, g). The thickness of CNFs coated layer became larger with increasing its concentration. The surface of CNF-treated GF is rougher than that of untreated GFs (Fig. 4a), increasing the specific surface area. This suggests the possibility of mechanical interlocking between GFs and epoxy matrix. In Fig. 4c–e, thinner CNF layers without aggregates can be observed, indicating a good dispersion of CNFs and higher porosity, which may give rise to better resin impregnation. However, when 0.1 wt% of CNFs was sprayed, the CNF layer became thicker and denser. The density of the CNF layer may prevent the epoxy resin from impregnating the reinforcing fibers, which might lead to a low interfacial adhesion between GFs and epoxy. It was found that the densest CNF layer indicated lower porosity making resin impregnation difficult [28]. Moreover, CNF aggregations are formed, making heterogeneous dispersion over the GF surface.

### 3.2 Interlaminar fracture toughness G_IIC_

**Fig. 5:**
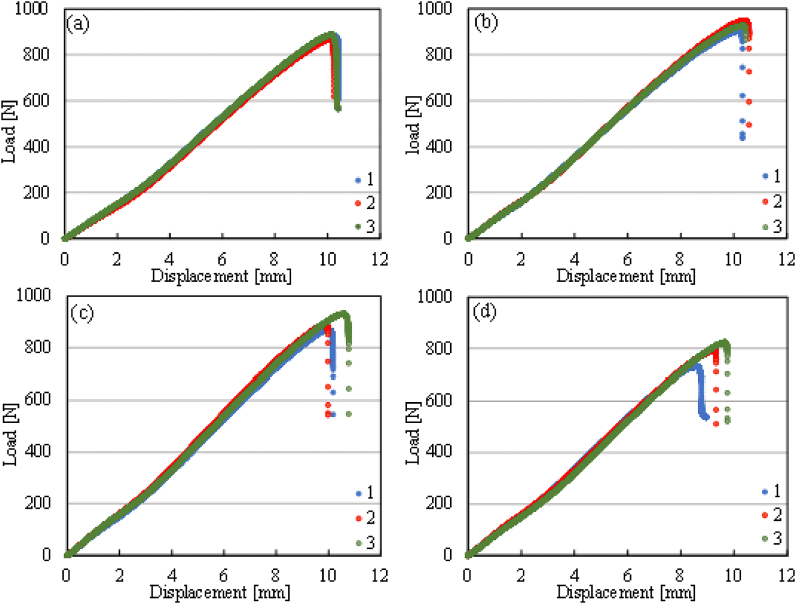
Load vs. displacement curves of the neat GF/EP laminate (a) and GF-CNFs/EP laminates for 0.05 wt% (b), 0.075 wt% (c), and 0.1 wt% (d) from ENF data.

The interlaminar fracture toughness of GFRP laminates was investigated under mode II loading. Fig. 5 shows load vs. displacement curves for neat glass fiber/epoxy (GF/EP) composite and CNFs-treated glass fiber/epoxy (GF-CNFs/EP) composite laminates. The load increased with the displacement showing a linear relationship and stable crack propagation. The crack propagation started from the nonlinear point in the load vs. displacement curves. When the load reached the critical value, unstable crack propagation occurred, and the load decreased. It is shown that the load for GF-CNFs/EP composites, except 0.1 wt % CNF (Fig. 5d), increased from 881 up to 926 N indicating better resistance to crack propagation and delamination (see Fig. 5b-c). Almost the same slope can be observed from the curves because CNFs did not affect the thickness and density of GF-CNFs/EP composites. The crack initiation of interlaminar fracture toughness (G_IIC, initiation_) is determined by appraising the fracture energy rate from the load at the nonlinear ($P_{NL}$) point from load-displacement curves. The crack propagation of interlaminar fracture toughness (G_IIC, propagation_) of GRFP composites is evaluated by calculating the critical energy release rate from the maximum load ($P_{c}$).

**Fig. 6:**
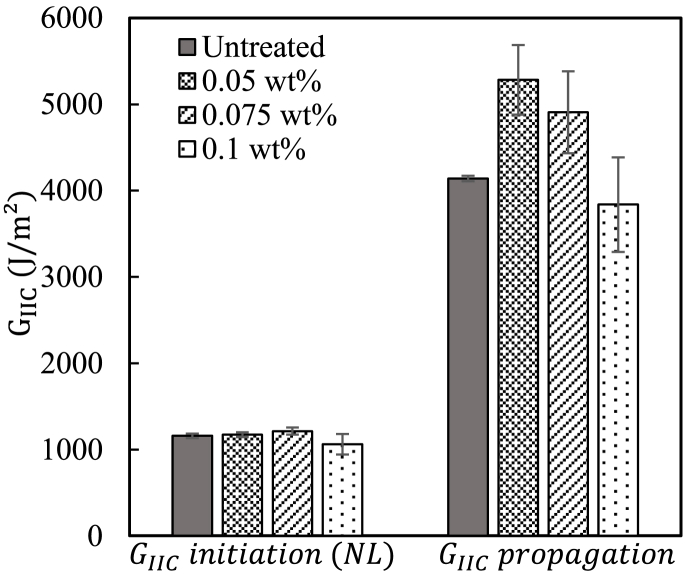
Interlaminar fracture toughness GIIC of neat GF/epoxy and GF-CNFs/EP composites.

Energy release rates G_IIC_ for crack initiation and crack propagation were calculated using Eqs (1) and (2). Fig. 6 shows the average of the interlaminar fracture toughness of GFRP composites. It can be easily seen that the G_IIC_ of GF-CNF/EP composites (up to 0.075 wt%) is higher than that of the neat GF/EP composite. After spraying 0.05 and 0.075 wt% of CNFs, G_IIC, initiation_ increased slightly due to the low CNF content while G_IIC, propagation_ showed significant improvements. The G_IIC, propagation_ of the GF-CNFs/EP composites with 0.05 and 0.075 wt% improved by 28% and 19%, respectively, compared to the GF/EP composite. The maximum improvement of G_IIC, propagation_ obtained from the composite with 0.05 wt% CNFs can be ascribed to the thin CNF nanostructures allowing a good impregnation of fibers with epoxy matrix.

In comparison, 0.075 wt% of CNFs exhibits a slight decrease in comparison with 0.05 wt%, which may result from the increase of CNF coating layers. However, these improvements in G_IIC_ can be attributed to the stronger interfacial bonding of GF-CNFs/EP composites compared to the neat GF/EP composite. The web-like CNF nanostructure observed in Fig. 4 might also contribute to toughening epoxy resin, indicating a better fracture toughness of the composites. On the other hand, a degradation of the interlaminar fracture toughness is exhibited at 0.1 wt% due to the thicker CNF coating layers, which may prevent epoxy resin from impregnating the reinforcing GF. With this CNF concentration, G_IIC, propagation_ decreases from 4141 to 3838 J/m^2^ indicating a weak GF-CNFs/EP adhesion.

The low energy release rate at crack initiation G_IIC, initiation_ can also result from the high loading speed as reported in a study conducted by De Baere et al. [29], where two crosshead speeds (0.5 and 1 mm/min) were used. These results revealed that the specimen loaded with high speed resulted in lower crack initiation and higher crack propagation, compared to that with low loading speed. The same trend was also found by Berger et al., who studied the effect of loading rate on the mode II interlaminar fracture toughness of CF/PEEK composites [30].

It is well known that the presence of CNFs on the GF surface increases the surface area, which induces mechanical interlocking between GF and EP. This can contribute to improving the interlaminar fracture toughness due to the irregular path of crack [8]. In the study by Wang et al. [9], CNCs-modified polyetherimide (PEI) nanofibrous interleaves were used to improve the interlaminar fracture toughness of CFRP composites. The mode II fracture toughness has been enhanced up to 20% with 6 wt% CNC. Moreover, the addition of MWCNT + n-butyl glycidyl ether into the epoxy matrix of GFRP resulted in increasing the interlaminar fracture toughness by about 23%. Interestingly, the result of this work exhibits higher improvement in G_IIC_ with a very low CNF concentration (e.g., 0.05 wt%) compared to the result obtained by Wang et al. The same comparison can also be made with the results of Zhu et al. [8] where a maximum improvement of 25% in G_IIC_ was reported with the same CNF concentration. On the other hand, the use of CNF with chopped flax fibers (FF) as interlayer resulted in higher improvement of about 100% in G_IIC_ of CF/EP composites owing to the good compatibility between CNFs and FF [28]. Therefore, the compatibility of CNF with reinforcing fibers plays an essential role in its dispersity and resin impregnation.

### 3.3 Fractography

**Fig. 7:**
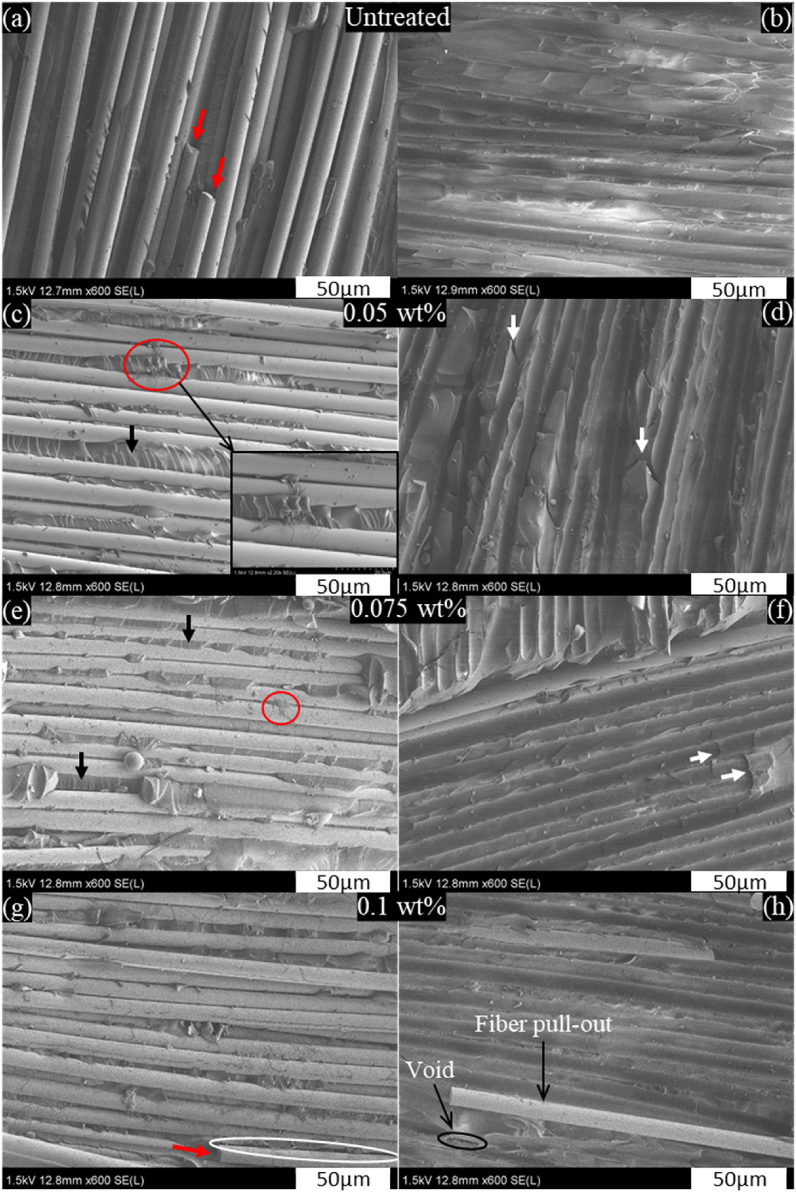
FE-SEM images of fractured surfaces after ENF test for: neat GF/EP composite (a–b), and GF-CNF/EP composites with 0.05 wt% (c–d), 0.075 wt% (e–f), 0.1 wt% (g–h).

The fracture surfaces of the GF/EP and GF-CNF/EP composites are presented in Fig. 7 to better understand the toughening mechanisms of delamination. The SEM images reveal that the GF surfaces of GF-CNF/EP composites are rougher than those of neat GF/EP composite. Fiber breakage and smooth traces of fiber pull-out from interfacial debonding can be observed in Fig. 7a and b, corresponding to low interfacial adhesion between GF and EP. In Fig. 7c–e, residual epoxy and CNFs-EP (in red circles) fragments remain attached on the GFs surface, indicating a good fiber/epoxy interface bond. In addition, shear hackles between fibers (in black arrows) and large epoxy deformations (in white arrows) can be seen in Fig. 7c–e and d-f, respectively. However, epoxy deformations are more frequent in the 0.05 wt% composite than those in the 0.075 wt% composite laminate. Shear hackles are also more obvious in Fig. 7c, which can be ascribed to stronger interlaminar shear strength. The increased fracture surface of hackles creates more energy dissipation and hence improves interlaminar fracture toughness [3,31]. The mechanisms aforementioned are attributed to shear stress due to the presence of CNFs at the mid-plane of GFRP composites indicating better resistance of interlaminar fracture toughness. It was found that adding CNFs onto the reinforcing fiber surfaces increases the specific surface area, improving the interfacial bonding and mechanical friction between fibers and epoxy [28]. Furthermore, CNF bridging was revealed to be effective in dissipating the fracture energy of delamination in a specimen under shear loading and hindering crack propagation. On the other hand, the improvement of the interlaminar toughness of composites with nanoparticles required the control of the orientation and the distribution of the nanoparticles leading to two main toughening mechanisms: the bridging and debonding of the nanoparticles [32]. Similarly, Prasad et al. [33] improved significantly the interlaminar fracture toughness mode I and mode II of flax fiber/epoxy composites by matrix modification with nano titanium dioxides. The highest improvement in fracture toughness mode II was attributed to the toughness and roughness of the matrix. Therefore, the roughness of the matrix at the interlayer of composite laminates contributes to improving interlaminar fracture toughness.

In contrast, we can observe in Fig. 7g large fiber/epoxy debonding (in white circle) and fiber breakage (in red arrow), arising from low fiber/epoxy interfacial bond due to the thicker CNF coating layer. Fig. 7h indicates smoother fiber pull-out traces and large void (in black circle) compared to 0.05 wt% GF-CNFs/EP composite (Fig. 7d). Despite the matrix deformation, reducing fiber/matrix interfacial strength decreases resistance crack propagation [34]. Epoxy matrix micro-cracking in composite contributed to a much lower G_IIC_ propagation. Therefore, these mechanisms could explain the reason for the low interlaminar fracture toughness in 0.1 wt% GF-CNFs/EP composite. Moreover, Fig. 9 illustrates the crack propagation path at the interlaminar fracture toughness of the composite laminates. The path propagation of crack for the untreated composite laminate is regular resulting from a weak interlaminar adhesion interface. Meanwhile, at 0.05 wt% crack exhibited irregular propagation, which may delay the crack propagation.

**Fig. 8:**
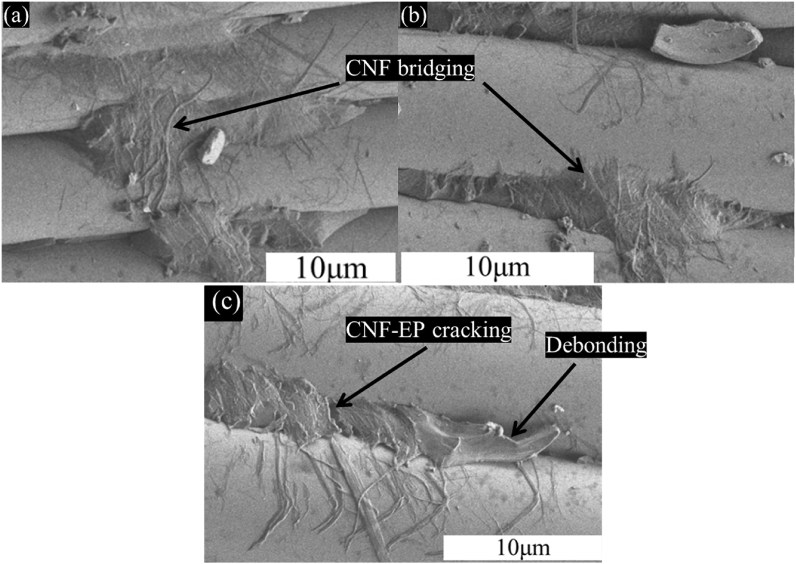
Fracture surface of GF-CNFs/EP composites at higher magnification for the toughening mechanisms of CNFs with: 0.05 wt%: (a), 0.075 wt%: (b) and 0.1 wt%: (c).

Fig. 8 shows more detailed information to clarify the interlaminar toughening mechanisms of CNFs at the GF/EP interface. CNF bridging and CNFs-EP residuals with perfect interfacial bonding observed on the fiber surface are resulted from the complete impregnation of resins. This because, the weblike structure observed in Fig. 4c and e contributed to the formation of tougher CNFs-EP matrix at the GF/EP interface. Hence, the friction between composite laminates is enhanced and shear hackles are increased leading to improving the interlaminar fracture toughness of the composites (see Fig. 8a and b). In contrast to 0.1 wt% of CNFs in Fig. 8c, CNFs-EP cracking and large debonding are observed between fibers. This may be attributed to the thicker layer of CNFs which prevented the impregnation of epoxy resin to fibers.

**Fig. 9:**
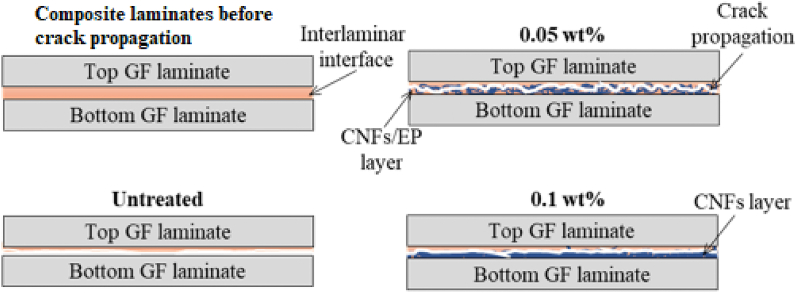
Illustration of crack propagation in the GFRP composite laminates.

**Fig. 10:**
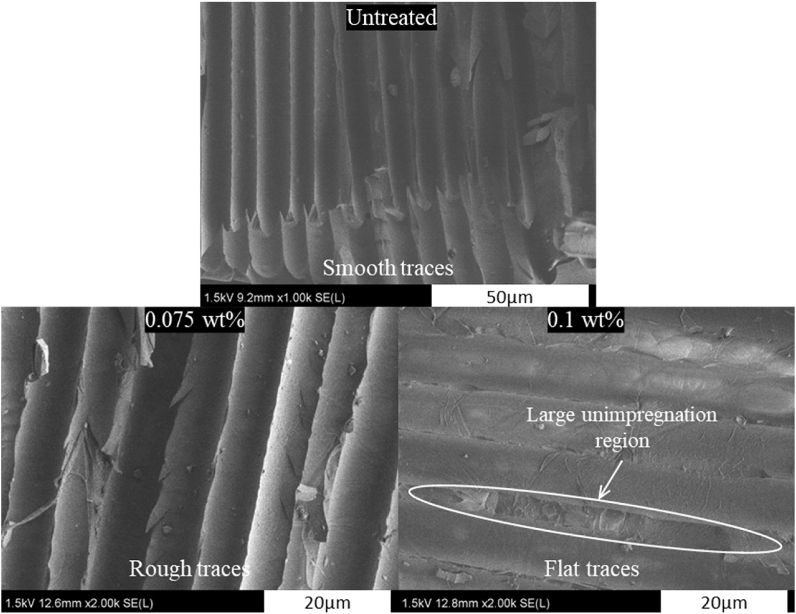
Fracture morphology of fiber traces at higher magnification for untreated, 0.075 and 0.1 wt% of GF/EP composites.

The images of fracture surfaces with high magnification were taken to understand further the mechanism for improving the interlaminar fracture toughness. Fig. 10 shows the fracture morphology of fiber traces after ENF tests to elucidate the toughening mechanisms. It can be seen that the neat GF/EP composite shows smooth traces from fiber pull-out without epoxy deformations. In the specimen with 0.075 wt% CNFs, fiber traces are rougher (with epoxy deformations) than the neat GF/EP composite, indicating strong bonding between GF-CNFs and EP. As for 0.1 wt% CNFs, flat fiber traces are observed resulting from resin unimpregnation regions caused by the bridging of thick and dense CNF layers between adjacent fibers. Hence, the interface between GF-CNFs and EP is very weak, decreasing the composite's interlaminar fracture toughness. Overall, the toughening mechanisms of interlaminar fracture toughness mode II of GF/EP composites by CNFs are dominated by CNF bridging, epoxy deformation and shear hackles.

## 4 Conclusion

This study proposed an alternative method of applying CNFs onto GFs to improve interlaminar fracture toughness mode II of GFRP composites. The effect of CNFs on interlaminar fracture toughness G_IIC_ of the modified GFRP composites was investigated. It was indicated that the presence of CNFs at the interlaminar interface of GF/EP laminate can improve the G_IIC_. Initiation of fracture toughness G_IIC initiation_ was moderately increased, whereas G_IIC propagation_ exhibited significant improvement. The optimum concentration was revealed to be 0.05 wt% with an improvement of 28% in G_IIC propagation_. However, the highest CNF concentration (0.1 wt%) led to a degradation in G_IIC_ due to the thickening of the CNF coating layer, which prevented epoxy resin from impregnating GFs. The toughening mechanisms such as epoxy deformation, CNF bridging, the roughness of fiber traces, and shear hackles are predominant for improving the interlaminar fracture toughness of GF-CNFs/EP composites.

## Author contribution statement

Mouhamadou Moustapha SARR: Conceived and designed the experiments; Performed the experiments; Analyzed and interpreted the data; Contributed reagents, materials, analysis tools or data; Wrote the paper.

Tatsuro KOSAKA: Conceived and designed the experiments; Contributed reagents, materials, analysis tools or data.

## Funding statement

This research did not receive any specific grant from funding agencies in the public, commercial, or not-for-profit sectors.

## Data availability statement

I have used the data in my PhD dissertation which will be publicly available repository after the acceptation and publication of the manuscript.

## Declaration of interest's statement

The authors declare no conflict of interest.
